# *Trans*-10, *cis*-12 conjugated linoleic acid reduces neutral lipid content and may affect cryotolerance of *in vitro-*produced crossbred bovine embryos

**DOI:** 10.1186/2049-1891-5-33

**Published:** 2014-06-10

**Authors:** Ribrio Ivan Tavares Pereira Batista, Nádia Rezende Barbosa Raposo, Paulo Henrique Almeida Campos-Junior, Michele Munk Pereira, Luiz Sergio Almeida Camargo, Bruno Campos Carvalho, Marco Antonio Sundfeld Gama, João Henrique Moreira Viana

**Affiliations:** 1 Federal University of Juiz de Fora, Juiz de Fora, MG 36036-900, Brazil; 2 Embrapa Dairy Cattle Research Center, Juiz de Fora, MG 36038-330, Brazil; 3 Federal University of Minas Gerais, Belo Horizonte, MG 31270-901, Brazil

**Keywords:** Blastocysts, CLA, Crossbred cattle, Cryopreservation, Lipid content

## Abstract

**Background:**

Due to high neutral lipids accumulation in the cytoplasm, *in vitro*-produced embryos from *Bos primigenius indicus* and their crosses are more sensitive to chilling and cryopreservation than those from *Bos primigenius taurus*. The objective of the present study was to evaluate the effects of *trans*-10, *cis-*12 conjugated linoleic acid (CLA) on the development and cryotolerance of crossbred *Bos primigenius taurus* x *Bos primigenius indicus* embryos produced *in vitro*, and cultured in the presence of fetal calf serum. Bovine zygotes (n = 1,692) were randomly assigned to one of the following treatment groups: 1) Control, zygotes cultured in Charles Rosenkrans 2 amino acid (CR2aa) medium (n = 815) or 2) CLA, zygotes cultured in CR2aa medium supplemented with 100 μmol/L of *trans-*10, cis-12 CLA (n = 877). Embryo development (cleavage and blastocyst rates evaluated at days 3 and 8 of culture, respectively), lipid content at morula stage (day 5) and blastocyst cryotolerance (re-expansion and hatching rates, evaluated 24 and 72 h post-thawing, respectively) were compared between groups. Additionally, selected mRNA transcripts were measured by Real–Time PCR in blastocyst stage.

**Results:**

The CLA treatment had no effect on cleavage and blastocyst rates, or on mRNA levels for genes related to cellular stress and apoptosis. On the other hand, abundance of mRNA for the 1-acylglycerol-3-phosphate 0-acyltransferase-encoding gene (AGPAT), which is involved in triglycerides synthesis, and consequently neutral lipid content, were reduced by CLA treatment. A significant increase was observed in the re-expansion rate of embryos cultured with *trans*-10, *cis-*12 CLA when compared to control (56.3 vs. 34.4%, respectively, *P* = 0.002). However, this difference was not observed in the hatching rate (16.5 vs. 14.0%, respectively, *P* = 0.62).

**Conclusions:**

The supplementation with *trans*-10, *cis-*12 CLA isomer in culture medium reduced the lipid content of *in vitro* produced bovine embryos by reducing the gene expression of 1-acylglycerol 3-phosphate 0-acyltransferase (AGPAT) enzyme. However, a possible improvement in embryo cryotolerance in response to CLA, as suggested by increased blastocyst re-expansion rate, was not confirmed by hatching rates.

## Background

Dairy herds in tropical countries are often composed of crossbred *Bos primigenius taurus* x *Bos primigenius indicus* cows in order to take advantage of heterosis and characteristics from zebu breeds such as resistance to heat stress [1]. *In vitro* embryo production (IVEP) technique has been largely used in tropical countries like Brazil to improve the genetic value for milk traits in Zebu breeds and to obtain crossbred Holstein-Zebu dairy cows [2]. However, crossbred *Bos primigenius taurus* x *Bos primigenius indicus* embryos produced *in vitro* are less cryotolerant when compared to *Bos primigenius taurus* embryos, due to increased lipid accumulation in blastomeres’ cytoplasm [3,4]. The low survival rate of those embryos after cryopreservation is a major obstacle to a wider use of IVEP and frozen embryos still represent less than 5% of all embryo transfers in Brazil, limiting the potential of *in vitro* embryo technology [2]. The high neutral lipid content, especially triglycerides stored in the lipid droplets present in the blastomeres [5], associated with the low concentration of polyunsaturated fatty acids in membrane phospholipids have been suggested as the major cause of low cryotolerance of *in vitro* produced embryos [6-8]. Excessive endogenous neutral lipid accumulation affects the equilibrium of dehydration and rehydration during embryo freezing and thawing [9], triggering ice crystals formation and thereby exacerbating the deleterious effects of cryopreservation.

The conjugated linoleic acid (CLA; generic term for a group of 18-carbon fatty acids with a conjugated double bond) has been shown to influence a range of biological processes, presenting anti-carcinogenic, antiatherogenic and anti-obesity properties. Specifically, the *trans*-10, *cis-*12 CLA isomer has been shown to alter membrane lipid composition [10], to inhibit lipid synthesis in different animals, and to regulate the expression of genes involved in the *de novo* fatty acids and triglycerides synthesis [11-13]. Additionally, supplementation of *in vitro* culture systems with *trans*-10, *cis-*12 CLA was suggested to improve bovine (*Bos primigenius taurus*) embryo survival after cryopreservation [14]. However, some studies with pre-neoplastic, neoplastic, and mammary gland cells have shown that *trans*-10, *cis-*12 CLA can also induce apoptosis, both by the endoplasmic reticulum (ER) pathway [15] and by the mitochondrial pathway [16], due to increased levels of free radicals and aldehydic products formed by the catabolism of this fatty acid [17]. Although these are different cell types, the early embryo cells show high metabolic activity, similarly to tumor cells [18], and it is possible that *trans*-10, *cis-*12 CLA causes some effects in the embryonic cells similar to those reported for somatic cells. The apparent contradiction between potentially beneficial and detrimental effects of *trans*-10, *cis-*12 CLA has not been addressed in previous studies evaluating embryos cultured in the absence of additional antioxidant protection, such as routinely used in the IVEP industry [17,19,20].

Eventual side effects of CLA can be evaluated by the analysis of the expression of genes related to cell stress and apoptosis, such as Peroxiredoxin (PRDX), heat shock proteins (HSP), and B-cell lymphoma 2 (Bcl-2) family. PRDXs are a ubiquitous family of thiol-specific antioxidant enzymes that control cytokine-induced peroxide levels, by reducing and detoxifying a wide range of organic hydroperoxides (ROOH) [21]. Hsp70s are an important part of the cell’s machinery for protein folding, and help to protect cells in stress situation. The accumulation of unfolded or misfolded proteins caused by oxidative stress induces a cellular protective response called “unfolded protein response”, characterized by increased expression of Hsp70 [22]. The B-cell lymphoma 2 family proteins control the release of cytochrome c during the apoptotic pathway. The proapoptotic proteins Bax and Bak form oligometric channels in the mitochondrial outer membrane, and cause the exit of the cytochrome c into the cytosol. In contrast, antiapoptotic family members Bcl-2 and Bcl-XL sequester the proapoptotic proteins, preventing the formation of protein-conducting channels and inhibiting apoptosis [23]. The proportion of the relative expression of Bax and Bcl-2 genes is commonly used as an indicator of apoptosis. However, Ip et al. [24] demonstrated that CLA induces apoptosis in mammary tumor cells by reducing the expression of Bcl-2, but without affecting the expression of proapoptotic members (Bak and Bax).

Thus, the aim of this study was to evaluate the effects of *trans*-10, *cis-*12 CLA in a conventional *in vitro* culture system on crossbred *Bos primigenius taurus* x *Bos primigenius indicus* embryos. The CLA effects were evaluated for embryo development rates, lipid accumulation, cryotolerance, abundance of mRNA for lipogenic enzymes such as acetyl-CoA carboxylase beta (ACACB), fatty acid synthase (FASN) and 1-acylglycerol 3-phosphate 0-acyltransferase (AGPAT1), as well as proteins involved in cell stress such as heat shock proteins 70.1 (Hsp70.1) and peroxiredoxin 1 (PRDX.1), and apoptosis, such as B-cell lymphoma 2 (Bcl-2).

## Methods

### Chemicals and reagents

All chemicals and reagents were purchased from Sigma Chemical Co. (St. Louis, MO, USA) unless otherwise stated.

### *In vitro* embryo production

Ovaries of dairy crossbred cows (*Bos primigenius indicus* x *Bos primigenius taurus, primarily Holstein x Gir*) were collected from slaughterhouse and transported to the laboratory in saline solution (0.9% NaCl) at 35°C containing antibiotic (0.05 g/L of streptomycin sulfate). Follicles of 3–8 mm were aspirated and cumulus-oocyte complexes (COCs) of grades I and II [25] were selected, and washed twice in medium TALP-HEPES [26]. Groups of 30–40 COCs were then placed into four-well plates (NUNC, Roskilde, Denmark) for *in vitro* maturation (IVM). Each well contained 400 μL of tissue culture medium (TCM–199, Gibco Life Technologies, Inc., Grand Island, NY, USA) supplemented with 20 μg/mL follicle stimulating hormone (FSH; Pluset, Serono, Italy), 0.36 mmol/L sodium pyruvate, 10 mmol/L sodium bicarbonate, 50 mg/mL streptomycin/penicillin and 10% estrous cow serum. The COC were incubated for 22–24 h at 38.5°C in a humidified atmosphere containing 5% CO_2_. For *in vitro* fertilization, frozen/thawed semen was centrifuged at 9,000 × g for 5 min in a Percoll (Nutricell Nutrientes Celulares, Campinas, SP, Brazil) discontinuous density gradient (45–90%) to obtain motile spermatozoa. The pellet was centrifuged again at 9,000 × g for 3 min in FERT-TALP medium [27]. Semen from the same bull (*Bos primigenius taurus*) was used throughout experiment. At the end of the maturation period, 25–30 COCs were co-incubated for 20 h with 2 × 10^6^ spermatozoa/mL in 100 μL drops of FERT-TALP medium supplemented with heparin (20 μg/mL) and bovine serum albumin (BSA) fatty acid free (6 mg/mL) under mineral oil. For embryo culture, presumptive zygotes were partially denuded by mechanically pipetting in TALP-HEPES medium and then randomly allocated to the experimental groups and co-cultured with their respective cumulus cells in four-well plates (NUNC, Roskilde, Denmark). Embryo culture was performed under the same atmospheric conditions of fertilization. After 72 h post-insemination (hpi) (d 3) 50% of the medium (CR2aa) was renewed and cleavage rate assessed. Blastocyst rate was assessed at 192 hpi (d8).

### Experimental design

After *in vitro* maturation and fertilization, presumptive zygotes (n = 1,692) were randomly assigned into two groups: Control – zygotes (n = 815) cultured in 500 μL CR2aa medium (modified from Rosenkrans Jr and First [28]) supplemented only with 10% FCS and 3 mg/mL BSA fraction V; or CLA – zygotes (n = 877) cultured in the same conditions of the control group, but with additional supplementation of the culture medium with 100 μmol/L *trans*–10, cis–12 CLA (Matreya, LLC, Pleasant Gap, PA, USA) as described by Pereira et al. [17]. Unlike Pereira et al. [17], however, in the present study no additional antioxidants were used in the culture medium. On the 3^rd^ day of culture (d 3) cleavage rate was assessed. On d 5, samples of embryos at morula stage (n = 15/group) were fixed for analysis of lipids content. On d 7, another sample of Grade I and II embryos at blastocyst stage (3 pools of 10 embryos/group) were placed in Eppendorf tubes and stored at – 80°C until RNA extraction. For both lipid content and gene expression analysis, embryos were sampled from different batches, and the remaining d 7 and d 8 Grade I and II blastocysts from all batches underwent slow freezing for analysis of pos-thawing survival rate or were used as controls (93 and 49 from control group, and 103 and 44 from the CLA group, respectively). Blastocyst rate was assessed on d 8 considering all blastocysts produced in drops where embryos were not removed for lipids quantification.

### Quantification of neutral lipid droplet content

Quantification of lipids was performed by Nile Red dye technique as described by Leroy et al. [29]. Briefly, d 5 embryos (n = 15 each groups) free of cumulus cells were fixed in a 500 μL of 2% glutaraldehyde and 2% formaldehyde solution at 4°C for at least 24 h. They were transferred to individual Eppendorf tubes (1 embryo/tube) containing 30 μL of a solution with 10 μg/mL Nile Red (Molecular Probes, Inc., Eugene, OR, USA) dissolved in saline solution (0.9% NaCl) with 1 mg/mL polyvinylpyrrolidone. Embryos were stained overnight in the dark and at room temperature. The Nile Red stock solution (1 mg/mL) was prepared previously with dimethyl sulfoxide (DMSO) and stored at room temperature in the dark. Final concentrations were obtained by diluting the stock with the saline solution. The amount of emitted fluorescent light of the whole embryo was evaluated at 582 ± 6 nm with an Olympus BX60 light microscope, equipped with an epifluorescence system using a 10 × objective. Images were captured separately using monochrome filters for FITC with a cooled charge-coupled device camera (Magnafire; Olympus). Images were imported into Adobe Photoshop (Adobe Systems, Mountain View, CA) as TIFF files. Fluorescence was quantified using the custom QUANTIPORO software (GNU Image Manipulation Program). The results were expressed in arbitrary units of fluorescence

### RNA extraction and relative quantification by real-time PCR

Total RNA extraction was performed from 3 pools of 10 embryos/group using RNeasy Micro kit (Qiagen, Hilden, Germany) according to manufacturer’s instructions and treated with DNase. Complementary DNA (cDNA) was synthesized using Superscript III First-strand supermix kit (Invitrogen, Carlsbad, CA, USA) and a random hexamer primer, according to manufacturer’s instructions. The cDNA quantification from each pool per group was performed using 1 μL of sample and a spectrophotometer ND-100 (NanoDrop Products, Wilmington, DE, USA). Relative quantification was performed in triplicate using Real-Time PCR (ABI Prism1 7300, Applied Biosystem, Forster City, CA, USA) and reactions using a mixture of Power SYBR Green PCR Master Mix (Applied Biosystem), 400 ng cDNA, nuclease-free water and specific primers for each reaction. Template cDNA was denatured at 95°C for 10 min, followed by 45 cycles of 95°C for 15 s; gene-specific primer annealing temperature for 30 s and elongation at 60°C for 30 s. After each PCR run, a melting curve analysis was performed to confirm that a single specific product was generated. Negative controls, comprised of the PCR reaction mix without nucleic acid, were also run for each group of samples. Amplicon size was confirmed by polycryalamide gel electrophoresis. Primer sequences and sizes of the amplified fragments for all transcripts are shown in Table 1. Primer efficiency was calculated for each reaction using Lin-RegPCR software [30]. The average efficiency of each set of primers was calculated and taking into account all groups. Expression of GAPDH gene was used as an endogenous reference. Relative abundance analyses were performed using REST software [31] and based on primer efficiency. Values found in embryos from CLA group are shown as n-fold differences relative to the control.

**Table 1:** Sequence of primers and annealing temperature specific for each gene

**Gene**	**Primer Sequence (5′ → 3’)**	**Annealing Temperature**	**Fragment Size**	**GenBank Accession Nº/Reference**
GAPDH*	F: CCAACGTGTCTGTTGTGGATCTGA	58°C	237	[42]
	R: GAGCTTGACAAAGTGGTCGTTGAG			
FASN	F – 5′ GCACCGGTACCAAGGTGGGC 3′	58°C	171	NM_001012669.1
	R – 5′ CGTGCTCCAGGGACAGCAGC 3′			
ACACB	F – 5′ CGGTGGTGCAGTGGCTGGAG 3′	58°C	254	XM_867921.3
	F – 5′ CAGGAGGACCGGGGGTCAGG 3′			
AGPAT1	F – 5′ CCGGAAGCGCACTGGGGATG 3′	58°C	170	NM_177518.1
	R – 5′ TGGGAACCTGGGCCTGCACT 3′			
PRDX1	F – 5′ ATGCCAGATGGTCAGTTCAAG 3′	53°C	224	[42]
	R – 5′ CCTTGTTTCTTGGGTGTGTTG 3′			
HSP70.1	F – 5′ AACAAGATCACCATCACCAACG 3′	59°C	275	NM_174550
	R – 5′ TCCTTCTCCGCCAAGGTGTTG			
BCL-2	F– 5′ TGGATGACCGAGTACCTGAA 3′	53°C	120	XM_586976
	R – 5′ CAGCCAGGAGAAATCAAACA 3′			

*Reference gene.

### Cryopreservation and Survival of embryos after thawing

Blastocysts classified as grade I and II according to Kennedy et al. [32] from both Control and CLA groups were washed in a solution of Dulbecco’s Phosphate-Buffered Saline (DPBS) with 0.4% BSA (Nutricell, Nutrientes Celulares, Campinas, SP, Brazil) and then dehydrated in a solution of 1.5 mol/L ethylene glycol in DPBS with 0.4% BSA (Nutricell, Nutrientes Celulares, Campinas, SP, Brazil), loaded into 0.25 mL straws and frozen in an automatic freezing device (Freeze Control, cryologic, Victoria, Australia) from – 6 to – 35°C at a 0.5°C per min rate. For thawing, each straw was kept 10 s in air and 20 s in a water bath at 35°C. Embryos were then co-cultured in CR2aa drops of 50 μL (15 embryos/drop) with a monolayer of granulosa cells for 72 h. The rate of re-expansion and hatching was assessed at 24 and 72 h of culture, respectively. The re-expansion and hatching rates were calculated based on the percentage of thawed embryos showing re-expansion of the blastocoel cavity and hatching of zona pellucida, respectively, after culture.

### Statistical analysis

The results of cleavage and blastocyst rates from each routine of IVEP were considered as replicates, while the lipid content was analyzed for each embryo. Data from IVEP and intensity of fluorescence were tested for normality and homocedasticity by Lilliefors and Cochran & Bartlett tests, respectively; after that, analysis of variance (ANOVA) was used to assess statistical differences. Values of relative expression of target genes were performed using the software REST using the Pair Wise Fixed Reallocation Randomization Test tool. Data for embryo survival after cryopreservation (re-expansion and hatching) were analyzed by chi-square method. The statistical significance was determined based on a *P*-value of 0.05.

## Results

### Developmental competence

Addition of *trans*-10, *cis-*12 CLA in culture medium did not affect cleavage (*P* = 0.06) and blastocyst rates (*P* = 0.20) as shown in Table 2. The developmental competence of cleaved embryos to reach blastocyst stage was also unaffected by CLA (44.2 and 46.2% for Control and CLA groups, respectively).

**Table 2:** **Development of bovine embryonic presumptive zygotes cultured in CR2aa medium supplemented with serum (Control) or with serum plus *trans*-10, *cis-*** **12 CLA**

**Treatment**	**Presumptive zygotes n**	**Cleavage d 3**	**Blastocyst d 8**
		**n (% ± SD)**	**n (% ± SD)**
Control	817	624 (74.5 ± 2.2)	276 (34.1 ± 2.6)
CLA	877	606 (70.3 ± 3.2)	280 (31.8 ± 2.4)

D3 = 72 h after fertilization, D8 = 192 h after fertilization, (n) = number of structures, SD = standard Deviation.

### Lipids quantification

**Figure 1:**
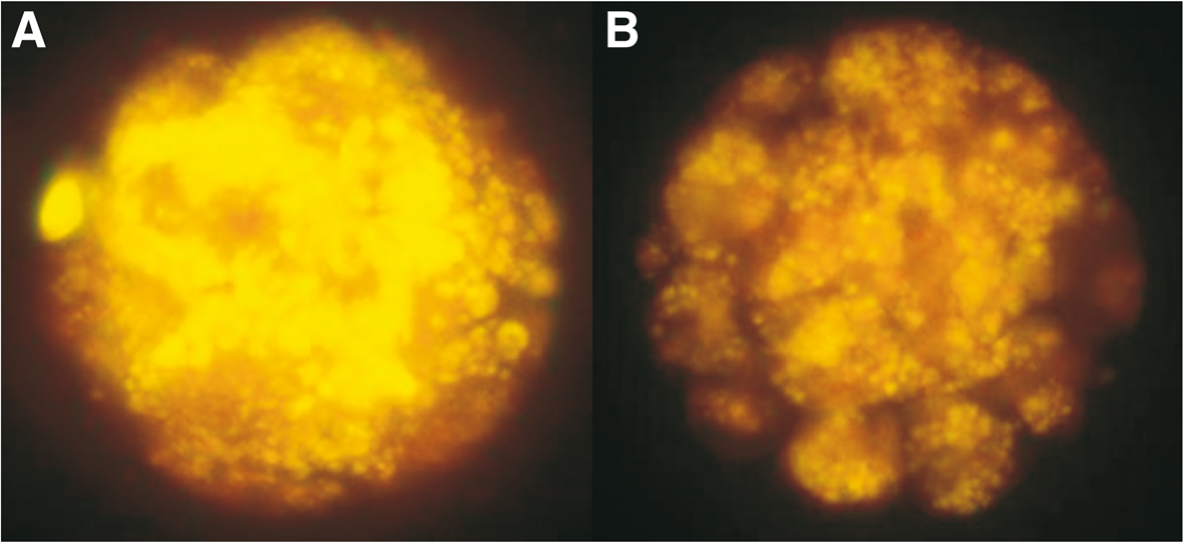
**Lipid content in embryos cultured in the presence of *trans*-10, *cis-*12 CLA.** Representative image of embryos produced *in vitro* and cultured in the absence **(A)** or presence **(B)** of *trans*-10, *cis-*12 CLA and stained with Nile Red die. The emitted fluorescence was restricted to lipid droplets (100 × magnification).

**Figure 2:**
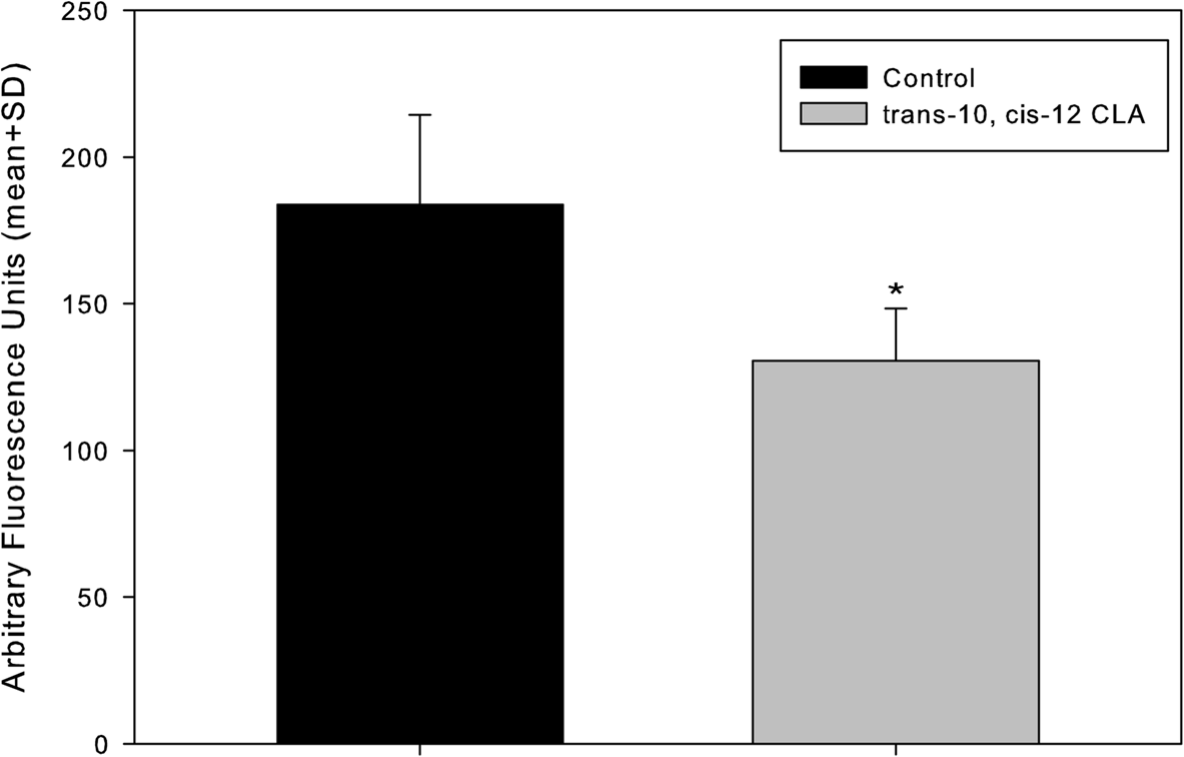
**Effect of *trans*-10, *cis-*12 CLA in embryo lipid accumulation.** Fluorescence emission (in arbitrary fluorescence units) of morula stage embryos cultured in the absence or presence of *trans*-10, *cis-*12 CLA. (*) Represents significant difference between the two groups (*P* = 0.0001).

Figure 1 shows the result of the fluorescence intensity of control and CLA embryos after Nile red staining. Morulas that were cultured in the absence of *trans*-10, *cis-*12 CLA contained more lipid droplets expressed as a significantly higher amount of emitted fluorescence light (*P* = 0.0001), compared with the morulas that were cultured in presence *trans*-10, *cis-*12 CLA (130.4 ± 30.5 vs. 183.9 ± 17.9 arbitrary fluorescence units, Figure 2).

### Analysis of target gene transcripts

**Figure 3:**
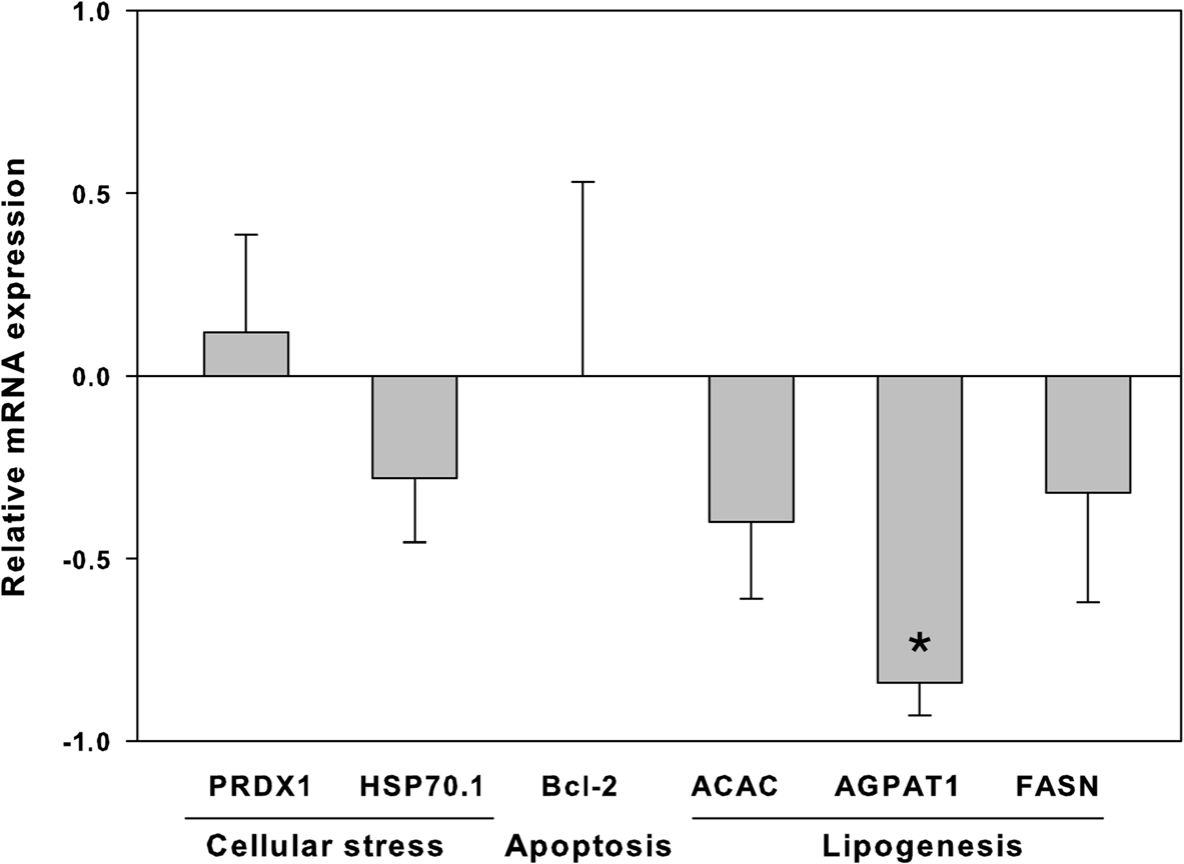
**Gene expression in embryos cultured in the presence of *trans*-10, *cis-*12 CLA.** Relative expression of transcripts of genes involved in cellular stress (PRDH1 and Hsp70.1), apoptotic process (Bcl-2), fatty acid synthesis (FASN and ACACB) and triglycerides synthesis (AGPAT1), through the Real - Time PCR in embryos cultured in the presence of *trans*-10, *cis-*12 CLA, in relation to the control group. (*) Represents significant difference between the two groups (*P* = 0.009).

Blastocysts cultured in CR2aa supplemented with 100 μM of *trans*-10, *cis-*12 CLA showed no effect (*P* > 0.05) on amount of mRNA expressed for Hsp70.1 (0.72 ± 0.17), PRDX1 (1.12 ± 0.27), and Bcl-2 (1.20 ± 0.26) genes when compared to the control group. Additionally, the analysis of transcripts of genes related to fatty acid synthesis showed a reduction (*P* = 0.009) in the amount of transcripts for AGPAT1 (0.16 ± 0.09) gene, but not for ACACB (0.6 ± 0.21, *P* = 0.18) and FASN (0.68 ± 0.30, *P* = 0.30) genes of blastocysts cultured with CLA (Figure 3).

### Survival of embryos after cryopreservation

There was a higher (*P* = 0.002) re-expansion rate after thawing of embryos cultured in medium supplemented with CLA (Table 3). This difference, however, was not reflected in a higher hatching rate (*P* = 0.62). Hatching rate in relation to re-expanded embryos was 43.75 and 29.31% for the control and CLA group, respectively. Fresh embryos from Control and CLA groups had similar (*P* = 0.16) hatching rate, which was higher than that of frozen-thawed embryos of Control and CLA groups.

**Table 3:** **Survival and developmental capacity after cryopreservation (as indicated by rates of re-expansion and hatching) of embryos cultured in CR2aa medium supplemented with serum (Control) or with serum plus *trans*-10, *cis-*** **12 CLA (CLA), compared to non-frozen embryos from the same groups**

**Group**	***In vitro *** **culture**	**Blastocyst**	**Re-expansion**	**Hatching**
		**n**	**% (n)**	**% (n)**
Control	Fresh	49	-	77.6 (38)^a^
CLA	Fresh	44	-	88.6 (39)^a^
Control	Frozen-thawed	93	34.4 (32)^a^	14.0 (13)^b^
CLA	Frozen-thawed	103	56.3 (58)^b^	16.5 (17)^b^

^a,b^Different lower case letters in the same column indicate statistical difference by Chi-square test (*P* < 0.01).

## Discussion

In the present study, we evaluated the effect of the addition of *trans*-10, *cis-*12 CLA in the culture medium of a conventional *in vitro* embryo production system. *In vitro* development of crossbred *Bos primigenius taurus* x *Bos primigenius indicus* embryos was not affected by supplementation of *trans*-10, *cis-*12 CLA in the absence of any additional antioxidant agent other than serum. CLA was suggested to increase survival rate after cryopreservation [17,22]. In these studies, however, culture media were added with antioxidant glutathione and β-mercaptoethanol, respectively, to prevent fatty acids oxidation. Moreover, in the study of Pereira et al. [17] media was renewed in a daily basis, while in the current study we target the potential effects of CLA in a conventional culture system, i.e., only one renewal of medium 72 h.p.i. Interestingly, the expression of gene transcripts of the PRDX.1 peroxiredoxin family member, which protect against the deleterious effects of oxidation in the cell, were unaffected by the CLA treatment. The catabolism of *trans*-10, *cis-*12 CLA occurs preferentially in peroxisomes [33]. In these organelles, the first reaction of β-oxidation produces H_2_0_2_ instead of FADH, as occurs in the mitochondria. Thus, the increase in PRDX1 expression by the embryo may occurs in response to the increased levels of H_2_0_2_ induced by the CLA. The results from the present experiment, however, suggest a low fatty acid metabolism during this period of development, which may have minimized the deleterious effects associated with the production of free radicals. Coherently, Thompson et al. [34] reported a reduction in the amount of ATP produced by the blastomeres, possibly due to a reduction in the proportional contribution of oxidative phosphorylation. This strategy may be required by the embryo due to the low concentration of oxygen in the way from oviduct to the uterus [35].

Similarly, the expressions of PRDX.1, cellular stress gene (Hsp70.1) and anti-apoptotic (Bcl-2) were not affected by fatty acid supplementation during embryo culture. Hsp70.1 is over-expressed in stress situations, such as elevated temperature, hypoxia, presence of reactive oxygen species, abnormal pH, and others, and has been shown to attenuate apoptosis [36], being frequently used as an indicator of stress in bovine embryos [1,37]. The Bcl-2, when down-regulated, allows the activation of Bax and thus the formation of protein channels through which pro-apoptotic factors such as cytochrome C are released into the cytosol, initiating the apoptotic cascade [38,39]. Although no effect of *trans*-10, *cis-*12 CLA was reported on levels of Bax or on translocation of Bax to the mitochondria, significant down-regulation occurred in Bcl-2 expression in mouse mammary tumor cells cultured with CLA [18].

*Trans*-10, *cis-*12 CLA modulates the amount of intracellular lipids through transcription mediators, as transcription factors sterol-regulatory element binding protein (SREBP) [11]. In the present study, *trans*-10, *cis-*12 CLA supplementation during embryo culture down-regulated the expression of AGPAT1 gene in blastocysts, when compared to the control group. This result was associated with a reduction in lipid accumulation and with an increase in re-expansion rate after cryopreservation in the CLA group. The expressions of enzymes related to de novo synthesis of fatty acids (ACACB and FASN), however, were not affected. Previous studies suggested that the route by which the embryo synthesizes triglycerides was preferably by the assembly of fatty acids internalized by the cell [40,41]. Coherently, Gutgesell et al. [42] showed that dietary *trans*-10, *cis-*12 CLA down-regulates gene expression of the proteins responsible for fatty acids uptake by the cells. Altogether, the results of the present study bring additional evidences that the mechanism by which CLA affect lipid accumulation in the embryo is by a reduction in the incorporation and/or assembly of triglycerides, rather than by inhibiting de novo synthesis.

Hatching rate is largely used as an indicator of embryo developmental potential after cryopreservation. In the current study, we observed that *trans*-10, *cis-*12 CLA supplementation in culture medium did not increase hatching rate after cryopreservation. This result suggests that the reduction of intracytoplasmic fat caused by CLA, regardless a potential positive effect on re-expansion after cryopreservation, was not sufficient to protect the embryo from later detrimental effects of cryopresenvation. Alternatively, it is also possible that CLA supplementation in culture medium may have affected the mechanisms associated with the enzymatic digestion of the zona pellucida by the trofectoderm, impairing hatching. In fact, the reduction in intracytoplasmic fat content by delipidation does not disturb preimplantation development of *in vitro*-fertilized embryos and is beneficial for embryo survival after cryopreservation, but may impair further embryo development [43]. The apparent contradiction between the beneficial effects of CLA (reduced embryo neutral lipid content, increased re-expansion after thawing) and the lack of difference in hatching rates, as observed in the current, suggests that further adjustments in CLA concentration are needed to balance the its potential detrimental effects and improve embryo cryotolerance.

## Conclusions

The use of *trans*-10, *cis-*12 CLA supplementation in a conventional *in vitro* embryo production system has no deleterious effects on blastocyst rate and thus can be used as an alternative to reduce embryo neutral lipid accumulation in crossbred zebu x *B. taurus* embryos. The mechanism by which *trans*-10, *cis-*12 CLA reduces the neutral lipid content of *in vitro* produced embryos involves a down-regulation in the expression of the 1-acylglycerol-3-phosphate 0-acyltransferase enzyme. However, a possible improvement in embryo cryotolerance in response to CLA, as suggested by increased blastocyst re-expansion rate, was not confirmed by hatching rates.

## Abbreviations

ACACB: Lipogenic enzymes as acetyl-CoA carboxylase beta; AGPAT1: 1-acylglycerol 3-phosphate 0-acyltransferase; Bcl-2: B-cell lymphoma 2; BSA: Bovine serum albumin; CLA: Conjugated linoleic acid; COCs: Cumulus-oocyte complexes; DPBS: Dulbecco’s Phosphate-Buffered Saline; ER: Endoplasmic reticulum; FASN: Fatty acid synthase; FCS: Fetal calf serum; HPI: After post-insemination; Hsp70.1: Heat shock proteins 70.1; IVEP: *In vitro* embryo production; PRDX.1: Peroxiredoxin 1.

## Competing interests

The authors declare that they have no competing interests.

## Authors’ contributions

RTPB carried out the experiment, participated in the data collection, data analysis, and drafted the manuscript. PHAC-J helped in the *in vitro* embryo production, in embryo cryopreservation, and in quantification of lipid contents. MMP helped in gene expression analysis. NRBR, MASG and LSAC contributed in the conception and design of the experiment, preparation of the manuscript, and acquisition of funding. BCC helped in data analysis and interpretation. JHMV contributed in the conception and design of experiment, analysis and interpretation of data, preparation of the final version of the manuscript, critical revision of the content and acquisition of funding. All authors approved the final version of the manuscript for publication.
